# Exposure to high levels of glucose increases the expression levels of genes involved in cholesterol biosynthesis in rat islets

**DOI:** 10.3892/etm.2014.1812

**Published:** 2014-06-26

**Authors:** YIXUAN SUN, YUQING ZHANG, NA LI, HUA ZHANG, LIBIN ZHOU, LI SHAO

**Affiliations:** 1 Department of Geratology, East Hospital, Shanghai Tongji University, Shanghai 200120, P.R. China; 2 Department of Endocrine and Metabolic Diseases, Shanghai Institute of Endocrine and Metabolic Diseases, Shanghai Clinical Center for Endocrine and Metabolic Diseases, Ruijin Hospital, Shanghai Jiao Tong University School of Medicine, Shanghai 200025, P.R. China; 3 Department of Gastroenterology, Central Hospital of Zibo, Zibo, Shandong 255036, P.R. China

**Keywords:** high glucose, pancreatic β-cell, nutrition adaptation, cholesterol biosynthesis

## Abstract

Cells continually adjust their gene expression profiles in order to adapt to the availability of nutrients. Glucose is a major regulator of pxancreatic β-cell function and cell growth. However, the mechanism of β-cell adaptation to high levels of glucose remains uncertain. To identify the specific targets responsible for adaptation to high levels of glucose, the differentially expressed genes from primary rat islets treated with 3.3 and 16.7 mmol/l glucose for 24 h were detected by DNA microarray. The results revealed that the expression levels of genes that encode enzymes required for *de novo* cholesterol biosynthesis [3-hydroxy-3-methylglutaryl-CoA synthase 1 (Hmgcs1), 3-hydroxy-3-methylglutaryl-CoA reductase (Hmgcr), mevalonate (diphospho) decarboxylase (Mvd), isopentenyl-diphosphate δ-isomerase 1 (Idi1), squalene epoxidase (Sqle) and 7-dehydrocholesterol reductase (Dhcr7)] were significantly increased in islets treated with high levels of glucose compared with those in the islets treated with lower glucose levels. Quantitative polymerase chain reaction further confirmed that glucose stimulated the expression levels of these genes in a dose- and time-dependent manner. A similar result was obtained in islets isolated from rats subjected to 12, 24, 48 and 72 h of continuous glucose infusion. It has previously been recognized that cholesterol homeostasis is important for β-cell function. The present study provides, to the best of our knowledge, the first evidence for the involvement of the *de novo* cholesterol biosynthesis pathway in the adaptation of rat islets to high levels of glucose *in vitro* and *in vivo*.

## Introduction

As a major global chronic health problem, type 2 diabetes affects 336 million people worldwide (1). Type 2 diabetes develops when pancreatic β cells fail to secrete enough insulin in the face of insulin resistance or increased insulin demand, most likely due to β-cell exhaustion (2). Therefore, it is critical to reveal the mechanism of β-cell compensation and prevent the progression of β-cell decompensation for the long-term management of the disease.

Cells continually adjust their gene expression profiles in order to adapt to the availability of nutrients such as glucose (3,4). The majority of nonproliferating, differentiated cells depend on the efficiency of ATP production through oxidative phosphorylation to maintain their integrity. These cells metabolize glucose to pyruvate through glycolysis following which they oxidize the majority of the pyruvate to CO_2_ through the tricarboxylic acid (TCA) cycle in the mitochondria (5). In lipogenic tissues or rapidly proliferating cells, citrate, generated by the TCA cycle from glucose, is preferentially exported from the mitochondria to the cytosol and cleaved by ATP citrate lyase to produce cytosolic acetyl coenzyme A (acetyl-CoA) (6–8), which is a vital building block for the *de novo* biosynthesis of fatty acids and cholesterol. Previous studies have reported that islets from mice with a specific inactivation of the ATP-binding cassette transporter 1 (Abca1), a cellular cholesterol transporter, in their β cells, demonstrated altered cholesterol homeostasis and impaired insulin secretion (9–11). This suggests that abnormality of the cholesterol metabolism may contribute to the impaired β-cell function in diabetes. However, as glucose is a major regulator of pancreatic β-cell function and the main source of precursors (including acetyl-CoA) for cholesterol synthesis, whether high levels of glucose regulate the expression levels of genes responsible for *de novo* cholesterol biosynthesis in islets has not yet been validated.

## Materials and methods

### Materials

RPMI-1640 medium, fetal bovine serum and other culture reagents were obtained from Gibco Life Technologies (Grand Island, NY, USA). The cell culture plates were purchased from Nalge Nunc International (Roskilde, Denmark). Collagenase type XI was purchased from Sigma (St. Louis, MO, USA). The anti-3-hydroxy-3-methylglutaryl-CoA reductase (Hmgcr) antibody was purchased from Abcam (Cambridge, MA, USA). Anti-rabbit IgG conjugated with horseradish peroxidase was obtained from Cell Signaling Technology, Inc. (Beverly, MA, USA).

### Rat infusions

The animal treatment was reviewed and approved by the Animal Care Committee of Shanghai Jiao Tong University School of Medicine (Shanghai, China). Male Sprague Dawley rats (Shanghai Laboratory Animal Center, Shanghai, China), weighing 250–300 g, were housed under a controlled temperature (21°C) and a 12 h light-dark cycle with unrestricted access to water and a standard laboratory diet. The animals were randomly divided into two groups, 4 rats in each group, receiving either saline or glucose, with heparin (40 U/ml). The infusion technique was similar to that described by Bonner-Weir *et al* (12). Under general anesthesia, indwelling catheters were inserted into the right jugular vein. The catheters were tunneled subcutaneously and exteriorized at the base of the neck. Following a recovery period of three to five days, the rats were infused (via the jugular vein catheter at 2 ml/h) with either 0.45% saline or 50% glucose in 0.45% saline, for 12, 24, 48 and 72 h. Animals were allowed access to food and water *ad libitum* during the infusion period. Upon completion of the infusion, the animals were sacrificed and their islets were isolated. The isolated islets were stored at −80°C with TRIzol (Invitrogen Life Technologies, Carlsbad, CA, USA) until RNA isolation.

### Islet isolation and treatment

Islets of Langerhans were isolated from male Sprague Dawley rats by an *in situ* infusion of the pancreas with collagenase, and separated by density gradient centrifugation (13). The concentration of collagenase type XI was 0.5 mg/ml. Isolated rat islets were transferred to 6-well plates (300 islets per well) and cultured for 6, 12, 24, 48 or 72 h in RPMI-1640 medium containing 3.3, 8.3, 11.1 or 16.7 mmol/l glucose, supplemented with 0.25% bovine serum albumin (BSA) at 37°C and with 5% CO_2_.

### RNA sample preparation and array hybridization

Total RNA was extracted from isolated islets using TRIzol according to the manufacturer’s instructions. Sample labeling and array hybridization were performed according to the instructions for the Agilent One-Color Microarray-Based Gene Expression Analysis protocol (Agilent Technologies, Inc., Santa Clara, CA, USA). The total RNA from each sample was linearly amplified and labeled with Cy3-CTP (Agilent Technologies, Inc., Santa Clara, CA, USA). The labeled cRNAs were purified using an RNeasy Mini kit (Qiagen, Hilden, Germany). The concentration and specific activity (pmol Cy3/μg cRNA) of the labeled cRNAs were measured by NanoDrop (ND-1000; Thermo Scientific, Wilmington, DE, USA). A total of 1 μg of each labeled cRNA was fragmented by adding 11 μl of 10X blocking agent and 2.2 μl of 25X fragmentation buffer (both from Agilent Technologies, Inc.,). The mixture was heated at 60°C for 30 min and 55 μl 2X gene expression (GE) hybridization buffer (Agilent Technologies, Inc.,) was added to dilute the labeled cRNA. A total of 100 μl of the hybridization solution was dispensed into a gasket slide and assembled to the gene expression microarray slide. The slides were incubated for 17 h at 65°C in an Agilent Hybridization Oven (Agilent Technologies, Inc.). The hybridized arrays were washed and scanned using the Agilent DNA Microarray Scanner system (part no. G2565BA; Agilent Technologies, Inc.).

### Microarray data analysis

Agilent Feature Extraction software (version 11.0.1.1; Agilent Technologies, Inc.) was used to analyze the acquired array images. Quantile normalization and subsequent data processing were performed using the GeneSpring GX software package (version 11.5.1; Agilent Technologies, Inc.). Differentially expressed genes were identified through volcano plot filtering. Gene ontology (GO) and pathway analyses were performed using the standard enrichment computation method.

### Quantitative polymerase chain reaction (qPCR)

The total RNA isolated from the islets was reverse-transcribed using a Promega Reverse Transcription kit (Promega Corporation, Madison, WI, USA). In order to quantify the transcript abundance of the genes of interest, qPCR was performed using SYBR Green Premix Ex Taq (Takara Bio, Inc., Shiga, Japan) with a 7300 Real-Time PCR machine (Applied Biosystems, Foster City, CA, USA). The relative expression levels were normalized to 18S mRNA levels in each sample. The sequences of the specific primers that were used were as follows: 3-hydroxy-3-methylglutaryl-CoA synthase 1 (Hmgcs1), 5′-GTCCCTCCACAAATGACCAC-3′ (forward), 3′-ATG ACAGCCGACTCAGGTTC-5′ (reverse); Hmgcr, 5′-TGCTGCTTTGGCTGTATGTC-3′ (forward), 3′-TGAGCG TGAACAAGAACCAG-5′ (reverse); mevalonate kinase (Mvk), 5′-TGGAGCAACTGGAGAAGCTG-3′ (forward), 3′-ATGTCCAGGCTTGGGAGTGT-5′ (reverse); phosphomevalonate kinase (Pmvk), 5′-TCAGCTGTAGGCCTGGTG AA-3′ (forward), 3′-TGCTCCTTGAGTGGACCAGA-5′ (reverse); mevalonate (diphospho) decarboxylase (Mvd), 5′-GAAAACTTCGCTGGCTACGG-3′ (forward) and 3′-CAACCCCTTTCTCCAGATGC-5′ (reverse); isopentenyl diphosphate δ-isomerase 1 (Idi1), 5′-CACTGG CAGGAGTGATTGGA-3′ (forward), 3′-TTGCTGGCATTG ATTTCAGG-5′ (reverse); farnesyl diphosphate farnesyl transferase 1 (Fdft1), 5′-ACATGGCCATCAGTGTGGAG-3′ (forward), 3′-AATTCTGCCATCCCACATCC-5′ (reverse); squalene epoxidase (Sqle), 5′-TGGTGGAGGAATGACAGT CG-3′ (forward), 3′-AAGCAAGCTTTTCGGAGCTG-5′ (reverse); 7-dehydrocholesterol reductase (Dhcr7), 5′-CGC TTCCAAAGCCAAGAATC-3′ (forward), 3′-ACACAA TGAACGGTGCGAAG-5′ (reverse); sterol regulatory element binding protein 1 (Srebp1), 5′-CATCCGCTT CTTACAGCACA-3′ (forward), 3′-TCATGCCCTCCA TAGACACA-5′ (reverse); 18S, 5′-CACGGGTGACGGGGA ATCAG-3′ (forward), 3′-CGGGTCGGGAGTGGGTAA TTTG-5′ (reverse).

### Western blot analysis

The cultured islets in the 6-well plates were washed twice with ice-cold phosphate-buffered saline (PBS) and immediately placed into a lysis buffer containing 25 mmol/l 4-(2-hydroxyethyl)-1-piperazineethanesulfonic acid (HEPES; pH 7.4), 1% Nonidet P-40, 100 mmol/l NaCl, 2% glycerol, 5 mmol/l NaF, 1 mmol/l ethylenediamine tetraacetic acid (EDTA), 1 mmol/l Na_3_VO_4_, 1 mmol/l sodium pyrophosphate (NaPPi), 1 mmol/l phenylmethylsulfonyl fluoride, 10 μg/ml aprotinin, 5 μg/ml leupeptin and 5 μg/ml pepstatin. Lysates were centrifuged at 14,000 × g for 10 min at 4°C. The protein concentration of the extracts was determined according to the Bradford method, using BSA as the standard. Samples were separated by sodium dodecyl sulfate polyacrylamide gel electrophoresis (SDS-PAGE) on 8% polyacrylamide gels and transferred to polyvinylidene fluoride (PVDF)-Plus membranes (Bio-Rad, Hercules, CA, USA). The transferred membranes were incubated with anti-Hmgcr antibody at 1:1,000 dilution overnight at 4°C. Primary antibodies were detected with donkey anti-rabbit IgG conjugated with horseradish peroxidase at 1:2,000 for 1 h at room temperature. The blotted membrane was developed with enhanced chemiluminescence (ECL) Advance (Cell Signaling Technology, Inc., Boston, MA, USA) and imaged with a LAS-4000 Super CCD Remote Control Science Imaging system (Fujifilm, Tokyo, Japan).

### Statistical analysis

Data are presented as mean ± standard error of the mean. Comparisons were performed using analysis of variance (ANOVA) for multiple groups or the Student’s t-test for two groups. P<0.05 was considered to indicate a statistically significant difference.

## Results

### Genes involved in cholesterol biosynthesis are upregulated in rat islets exposed to high levels of glucose

**Figure 1:**
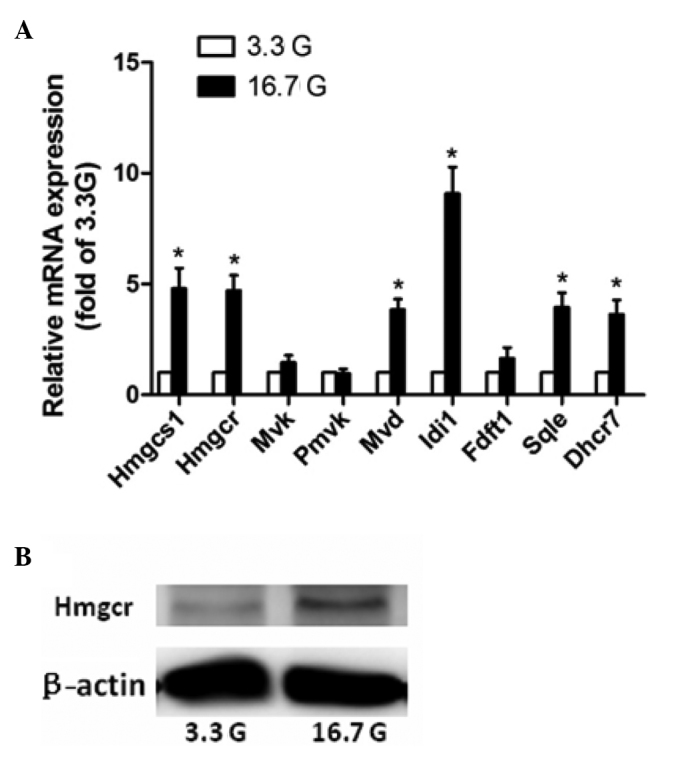
Effect of high levels of glucose on the expression levels of genes involved in cholesterol biosynthesis in rat islets *in vitro*. Isolated islets were incubated at 3.3 (3.3G) or 16.7 (16.7G) mmol/l glucose for 24 h. (A) The mRNA expression levels of nine genes involved in cholesterol biosynthesis were detected by quantitative polymerase chain reaction (qPCR). (B) The expression level of the 3-hydroxy-3-methylglutaryl-coenzyme A reductase (Hmgcr) protein was detected by western blot analysis. Data are expressed as mean ± standard error of the mean for three separate experiments. ^*^P<0.05 vs. 3.3 mmol/l glucose treatment. A representative blot from three independent experiments is shown. All three experiments revealed similar results. Hmgcs1, 3-hydroxy-3-methylglutaryl-CoA synthase 1; Mvk, mevalonate kinase; Pmvk, phosphomevalonate kinase; Mvd, mevalonate (diphospho) decarboxylase; Idi1, isopentenyl-diphosphate δ-isomerase 1; Fdft1, farnesyl-diphosphate farnesyl transferase 1; Sqle, squalene epoxidase; Dhcr7, 7-dehydrocholesterol reductase.

To identify the specific targets responsible for the adaptation to high levels of glucose, the differentially expressed genes from primary rat islets treated with 3.3 and 16.7 mM glucose for 24 h were detected by a whole-genome DNA microarray. Among a total of 44,000 probe sets, 894 genes were upregulated by high levels of glucose, revealing changes of ≥2-fold. GO analysis was used to identify the underlying biological themes in the genes in response to treatment with high levels of glucose. As shown in Table I, seven genes, termed in the gene ontology as ‘cholesterol biosynthetic process’ (GO: 0006695), were significantly upregulated in group treated with high levels of glucose. The qPCR results for Hmgcs1, Hmgcr, Mvd, Idi1, Sqle and Dhcr7 confirmed the genomic analyses, whereas those for Fdft1 did not (Fig. 1). Hmgcr, the rate-limiting enzyme in cholesterol biosynthesis, catalyzes the synthesis of mevalonate from hydroxymethyl glutaric acid acyl coenzymeA. Western blot analysis revealed increased protein levels of Hmgcr (Fig. 1). When combined, these data demonstrate that high levels of glucose markedly increased the expression levels of various genes involved in cholesterol biosynthesis in rat islets.

### Cholesterol biosynthetic gene expression levels are increased in the islets from rats infused with high levels of glucose

**Figure 2:**
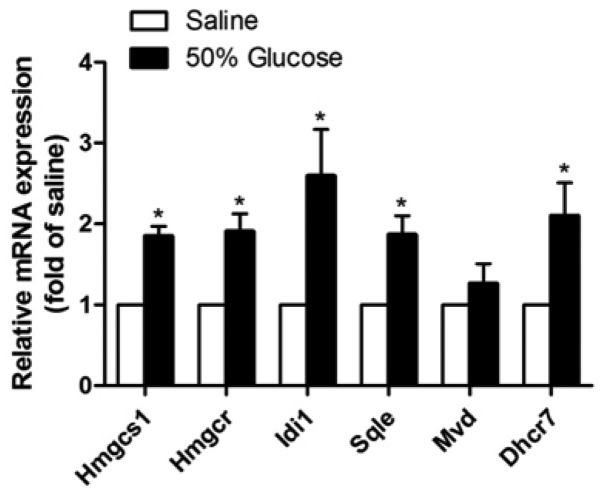
Infusion with high levels of glucose increases the expression levels of cholesterol biosynthesis genes in rat islets *in vivo*. Sprague Dawley rats were infused with 50% glucose or saline for 24 h (n=5). The islets were isolated and the expression levels of mRNA were detected by quantitative polymerase chain reaction (qPCR). Data are expressed as mean ± standard error of the mean. ^*^P<0.05 vs. saline treatment. Hmgcs1, 3-hydroxy-3-methylglutaryl-CoA synthase 1; Hmgcr, 3-hydroxy-3-methylglutaryl-CoA reductase; Idi1, isopentenyl-diphosphate δ-isomerase 1; Sqle, squalene epoxidase; Mvd, mevalonate (diphospho) decarboxylase; Dhcr7, 7-dehydrocholesterol reductase.

To further investigate the effect of high levels of glucose on cholesterol biosynthetic gene expression levels *in vivo*, the present study used a continuous glucose-infusion model as previously described (12). Infusion with 50% glucose (2 ml/h) for 24 h causes a significant increase in the plasma glucose level in rats (14,15). Consistent with the *in vitro* results of the current study, Hmgcs1, Hmgcr, Idi1, Sqle and Dhcr7 mRNA expression levels were significantly higher in islets isolated from rats infused with high levels of glucose for 24 h compared with those in the saline-infused control rats (Fig. 2). Furthermore, the expression level of the Idi1 gene revealed the highest increase (259±58%, P<0.05) among all genes tested, in accordance with *in vitro* results.

### Glucose increases the expression levels of cholesterol biosynthetic genes in islets in a dose-dependent manner

**Figure 3:**
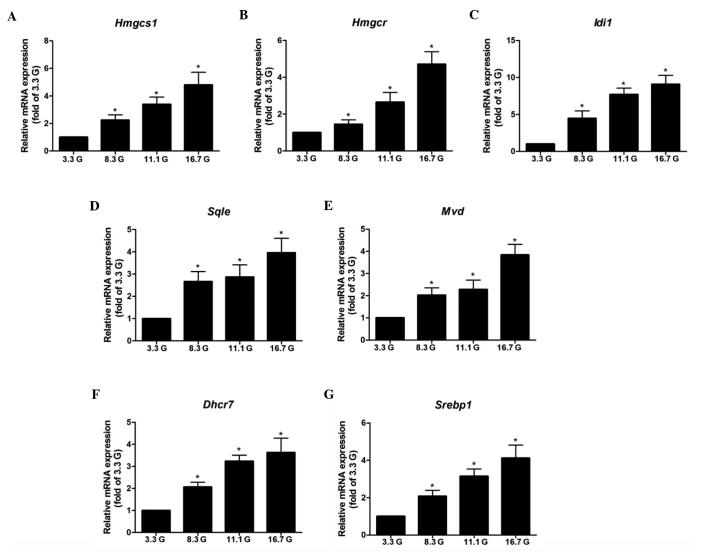
Dose-dependent effects of high levels of glucose on the expression levels of genes involved in cholesterol biosynthesis in rat islets. Following isolation, the rat islets were incubated with 3.3, 8.3, 11.1 or 16.7 mmol/l glucose and the expression levels of mRNA of (A) 3-hydroxy-3-methylglutaryl-CoA synthase 1 (Hmgcs1), (B) 3-hydroxy-3-methylglutaryl-CoA reductase (Hmgcr), (C) isopentenyl-diphosphate δ-isomerase 1 (Idi1), (D) squalene epoxidase (Sqle), (E) mevalonate (diphospho) decarboxylase (Mvd), (F) 7-dehydrocholesterol reductase (Dhcr7) and (G) sterol regulatory element-binding protein 1 (Srebp 1) were detected by quantitative polymerase chain reaction (qPCR). Data are presented as mean ± standard error of the mean. ^*^P<0.05 vs. 3.3 mmol/l glucose treatment.

Isolated rat islets were incubated in 3.3, 8.3, 11.1 and 16.7 mmol/l glucose for 24 h. The qPCR results demonstrated that glucose increased the mRNA expression levels of Hmgcs1, Hmgcr, Mvd, Idi1, Sqle, Dhcr7 and Srebp1 in a dose-dependent manner, with notable effects observed at the concentration of 8.3 mmol/l (Fig. 3). The expression level of the of Srebp1 gene, which functions as a transcription factor for the gene expression of cholesterol biosynthetic enzymes, was also assessed. The qPCR results revealed that glucose markedly enhanced the expression level of Srebp1 in the same pattern as its target genes (Fig. 3).

### Time course for the expression of cholesterol biosynthetic genes in islets exposed to high levels of glucose in vitro or in vivo

**Figure 4:**
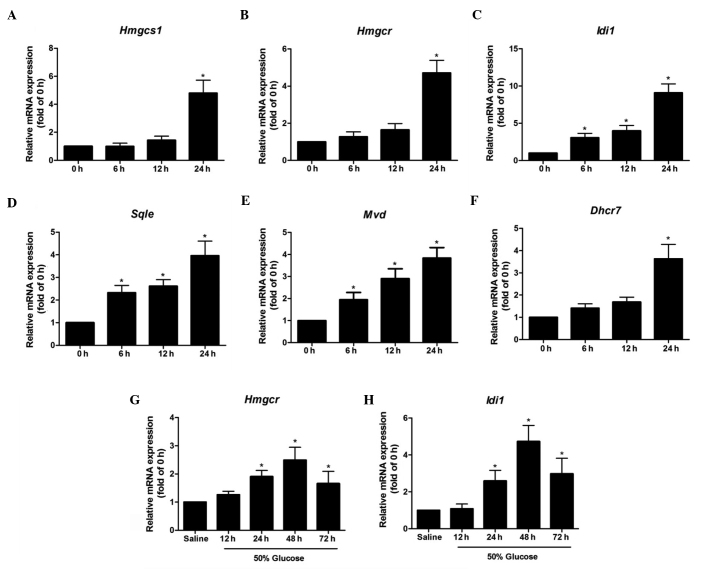
Time course for the expression of cholesterol biosynthetic genes in islets exposed to high levels of glucose *in vitro* or *in vivo.* Following isolation, the rat islets were incubated with 16.7 mmol/l glucose for the indicated time and the expression levels of mRNA of (A) 3-hydroxy-3-methylglutaryl-CoA synthase 1 (Hmgcs1), (B) 3-hydroxy-3-methylglutaryl-CoA reductase (Hmgcr), (C) isopentenyl-diphosphate δ-isomerase 1 (Idi1), (D) squalene epoxidase (Sqle), (E) mevalonate (diphospho) decarboxylase (Mvd) and (F) 7-dehydrocholesterol reductase (Dhcr7) were detected by quantitative polymerase chain reaction (qPCR). After the Sprague Dawley rats were infused with 50% glucose for the indicated time, the islets were isolated for analysis by qPCR to determine the mRNA expression levels of (G) Hmgcr and (H) Idi1. Data are expressed as mean ± standard error of the mean for three separate experiments. ^*^P<0.05 vs. 0 h or saline treatment.

The time-dependent effects of high levels of glucose on the expression levels of cholesterol biosynthetic genes in isolated rat islets were detected (Fig. 4). In islets exposed to 16.7 mmol/l glucose for 6 and 12 h, the mRNA levels of Hmgcs1, Hmgcr and Dhcr7 remained comparable with those at 0 h, and increased significantly at 24 h by 380±92, 371±68, and 263±65%, respectively (P<0.05). However, the gene expression levels of Sqle and Mvd were increased ~2-fold by high levels of glucose at 6 h, and increased markedly with the lengthening of the exposure time. The Idi1 gene exhibited the most notable change in expression level among the genes tested, increasing by 206±59% at 6 h and 808±119% at 24 h (P<0.05). In islets isolated from 50% glucose-infused rats for 12 h, the mRNA level of Hmgcr remained unchanged when compared with that of the saline control group; however, it significantly increased with the lengthening of the infusion time to 24 h, and reached a peak level after 48 h (Fig. 4). The expression level pattern of the Idi1 gene was similar to that of Hmgcr in islets from glucose-infused rats (Fig. 4).

## Discussion

As a main source for precursors (including acetyl-CoA), glucose has notable long-term actions on the *de novo* biosynthesis of lipids (16). Numerous studies investigating the link between chronic high levels of glucose and pancreatic β-cell lipid biosynthesis have focused on fatty acids (FAs). Chronic high levels of glucose markedly enhance the gene expression levels of acetyl-CoA carboxylase and fatty acid synthase and induce *de novo* fatty acid synthesis in pancreatic β cells (17–19). In the present study, the expression levels of genes involved in *de novo* cholesterol biosynthesis (Hmgcs1, Hmgcr, Mvd, Idi1, Sqle and Dhcr7) were significantly increased in islets treated with high levels of glucose. These results were further confirmed in islets isolated from rats subjected to 12, 24, 48 and 72 h continuous glucose infusion. This suggests that high levels of glucose may enhance cholesterol biosynthesis in the islets through increasing the gene expression levels of associated enzymes.

Cholesterol is synthesized via a cascade of enzymatic reactions known as the mevalonate pathway, which involves >20 enzymes from several subcellular compartments (20). This series of reactions is primarily regulated by a rate-limiting step involving the conversion of hydroxylmethylglutaryl-coenzyme A (HMG-CoA) into mevalonate. The rate-limiting reduction of HMG-CoA to mevalonate is an important regulatory step in cholesterol synthesis (21). The results of the present study revealed that high levels of glucose enhanced the gene expression levels of Hmgcr in rat islets in a dose-dependent manner when the cells were exposed to them for 24 h, and exposure to high levels of glucose also increased the protein levels of the gene. The qPCR results from glucose-infused rat islets confirmed this effect of high levels of glucose *in vivo*. Hmgcr acts early in the cholesterol synthesis pathway, with >20 subsequent enzymes required to produce cholesterol. The process of their regulation remains largely unstudied. The current study has provided the first published evidence, to the best of our knowledge, that high levels of glucose significantly increase the gene expression levels of Mvd, Idi1, Sqle and Dhcr7 in rat islets; these genes encode enzymes that serve as flux-controlling points in the cholesterol synthesis process beyond Hmgcr.

It has long been known that chronic high levels of glucose stimulate β-cell proliferation (12,22–26); however, the mechanism underlying the role of glucose in these events remains to be fully determined. Cell proliferation requires nutrients, energy and biosynthetic activity to duplicate all the macromolecular components during each passage through the cell cycle (27). The cholesterol content and rate of cholesterol biosynthesis are elevated in proliferating normal tissues and a reduction in cholesterol biosynthesis inhibits cell growth (28). This suggests the presence of a link between cell proliferation and the cholesterol biosynthetic pathway. The mevalonate pathway is also a crucial biochemical process for the generation of other key metabolic end products. There is an expanding list of intermediates that are known to be involved in cholesterol synthesis, and which have been credited with regulatory functions in the control of cell growth (28). Farnesyl diphosphate and other phosphorylated products of the mevalonate pathway are essential to the post-translational processing and physiological function of small G proteins, nuclear lamins and growth factor receptors (29–30), which are crucially involved in the regulation of proliferation. In addition, the expression levels of the steroidogenic acute regulatory protein (StAR) and steroid-5-α-reductase (Srd5a1), involved in the synthesis of steroid hormones from cholesterol, were observed to be increased by treatment with high levels of glucose in the DNA microarray data in the present study (data not shown). The regulatory mechanism of these genes by glucose and the possible role of these intermediates in glucose-stimulated β-cell proliferation requires further investigation.

In conclusion, based on the microarray analysis and qPCR validation, the present study identified a set of genes encoding cholesterol biosynthetic enzymes that were induced by high levels of glucose in rat islets. The current results provide evidence that enhanced cholesterol biosynthesis may play a role in the adaptive changes of β cells to high levels of glucose *in vivo* and *in vitro*.

## Figures and Tables

**Table I:** Increased expression folds of cholesterol biosynthesis-associated genes in isolated rat islets incubated with 16.7 compared with 3.3 mmol/l glucose as demonstrated by microarray analysis.

Gene symbol	Gene name	Gene function	mRNA fold change
Hmgcs1	3-Hydroxy-3-methylglutaryl- CoA synthase 1	Catalyzes the conversion of (S)-3-hydroxy-3-methylglutaryl-CoA and CoA to acetyl-CoA, acetoacetyl-CoA and H_2_O	2.494
Hmgcr	3-Hydroxy-3-methylglutaryl- CoA reductase	Enzyme involved in mevalonate synthesis	2.675
Mvd	Mevalonate (diphospho) decarboxylase	Enzyme that catalyzes the conversion of mevalonate pyrophosphate to isopentenyl pyrophosphate	2.044
Idi1	Isopentenyl-diphosphate δ-isomerase 1	Catalyzes the interconversion of isopentenyl diphosphate to dimethylallyl diphosphate	5.411
Fdft1	Farnesyl-diphosphate farnesyl transferase 1	Catalyzes the conversion of *trans*-farnesyl diphosphate to squalene	2.279
Sqle	Squalene epoxidase	Enzyme that catalyzes sterol biosynthesis	2.591
Dhcr7	7-Dehydrocholesterol reductase	Catalyzes the reduction of 7-dehydrocholesterol and is involved in cholesterol biosynthesis	2.139
Srebp1	Sterol regulatory element binding protein 1	Transcription factor: binds to the sterol regulatory element 1	2.289

## Abbreviations

TCA: tricarboxylic acid
BSA: bovine serum albumin
Hmgcs1: 3-hydroxy-3-methylglutaryl-CoA synthase 1
Hmgcr: 3-hydroxy-3-methylglutaryl-CoA reductase
Mvk: mevalonate kinase
Pmvk: phosphomevalonate kinase
Mvd: mevalonate (diphospho) decarboxylase
Idi1: isopentenyl-diphosphate δ-isomerase 1
Fdft1: farnesyl diphosphate farnesyl transferase 1
Dhcr7: 7-dehydrocholesterol reductase
Sqle: squalene epoxidase
Srebp1: sterol regulatory element binding protein 1
HMG-CoA: hydroxymethylglutaryl-coenzyme A
